# Molecular Cloning and Functional Characterization of Mouse α3(IV)NC1

**DOI:** 10.4137/cmo.s461

**Published:** 2008-02-09

**Authors:** Chandra Shekhar Boosani, Akulapalli Sudhakar

**Affiliations:** 1 Cell Signaling and Tumor Angiogenesis Laboratory, Department of Genetics, Boys Town National Research Hospital, Omaha, Nebraska, U.S.A; 2 Department of Biomedical Sciences, Creighton University School of Medicine, Omaha, Nebraska, U.S.A; 3 Department of Biochemistry and Molecular Biology, University of Nebraska Medical Center, Omaha, Nebraska, U.S.A

## Abstract

Non-collagenous α3 chain of type IV collagen or α3(IV)NC1, a 28 kDa C-terminal domain of collagen type IV is a specific inhibitor of endothelial cell translation and angiogenesis. In the present study we have cloned and expressed mouse α3(IV)NC1 in baculovirus system. The recombinant protein was expressed in soluble form and tested for several of its biological functions. We identified that this recombinant mouse α3(IV)NC1 specifically inhibited proliferation, translation and tube formation of endothelial cells. Also, we show that α3(IV)NC1 treatment results in apoptosis specifically in proliferating endothelial cells. In addition we report for the first time that mouse α3(IV)NC1 inhibits migration and p38 MAPK phosphorylation in addition to inhibition of FAK/Akt/mTOR/4E-BP1 signaling. In mice α3(IV)NC1 treatment reduced tumor growth and CD-31 positive endothelial vasculature in tumors. Collectively, our data demonstrate the expression of biologically active form of mouse α3(IV)NC1 in *Sf-9* cells and provide important mechanistic insights on α3(IV)NC1 antiangiogenic actions in endothelial cells.

## Introduction

The non-collagenous domain from the carboxy terminal region of α3 chain type IV collagen (α3(IV)NC1 or tumstatin), a specific inhibitor of endothelial cell proliferation and angiogenesis, was cloned as 28 kDa protein (Boosani and Sudhakr, 2007; Feng et al. 1994; Mariyama et al. 1994; Petitclerc et al. 2000). We have reported recently that human α3(IV)NC1 binds to α3β1/αVβ3 integrins and inhibits hypoxic cyclo-oxygenase-2 (COX-2) signaling leading to inhibition of tumor angiogenesis and tumor growth in mice (Boosani et al. 2007). The serum levels of circulating mouse α3(IV)NC1 was shown around 300 ng/ml, where as human endostatin was in a range between 120–300 ng/ml (Dhanabal et al. 1999; Hamano et al. 2003). The expression levels of human α3(IV)NC1 in mammalian system was reported to be very low (1–2 mg/liter) (Neilson et al. 1993). Based on our experience with these domains used in murine tumor studies, the amount of α3(IV)NC1 needed for preclinical use could not be obtained by mammalian expression system. Earlier researchers have cloned murine α3(IV)NC1 however, there was no clear evidence on its *in vitro* and *in vivo* functional characterization (Miner and Sanes, 1994). However, there was no clear evidence on its *in vitro* and *in vivo* functional characterization. We therefore cloned and expressed mouse α3(IV)NC1 using baculovirus expression system, in a manner similar to our recently reported work using human α1(IV)NC1(Boosani and Sudhakar, 2006).

In the present study, to understand biological functions of mouse α3(IV)NC1, the protein domain was cloned and expressed in *Sf-9* cells using the baculovirus expression system. The recombinant purified α3(IV)NC1 protein was found biologically active both *in vitro* and *in vivo,* as it inhibited endothelial cell proliferation and translation similar to human α3(IV)NC1. We show for the first time that mouse α3(IV)NC1 specifically inhibits endothelial cell migration and p38 MAPK phosphorylation in addition to inhibition of FAK/Akt/mTOR phosphorylation.

## Materials and Methods

Human umbilical vein endothelial cells (HUVEC) purchased from Clonetech^TM^ Inc; Martigel^TM^ Martix (14.6 mg/ml) was purchased from BD Biosciences Discovery lab. Recombinant human bFGF was purchased from R&D systems. Horseradish peroxidase (HRP)-labeled secondary antibodies, penicillin/streptomycin, fibronectin (FN), low melting Agarose and neutral red staining solutions were purchased from Sigma-Aldrich. Graces insect cell culture medium, cell fixer and H&E staining were purchased form Fisher Scientific Inc. Baculovirus transfer vector pAcHLT-A, transfection reagent and Affinity matrix (Ni-NTA Agarose) were purchased from PharMingen, USA. Caspase3 inhibitor DEVD was purchased from Chemicon; Protease solutions were purchased from Boehringer and Mannheim, GmbH. FCS, ECL Kit, Random primer labeling kit, hybond N^+^ membrane, (α-^32^P) dCTP, DNA ladder, competent DH5α cells, DNA polymerase 1, klenow fragment and Multiprime DNA labeling system were purchased from Amersham Biosciences. SuperScript one-step RT-PCR system and Lipofectamine Plus reagent obtained from Invitrogen. Restriction enzymes and *Pfx* polymerase were purchased from New England Biolabs.

## Cell Culture

Primary HUVECs were maintained in EGM-2 medium at 37 °C in a humidified 5% CO_2_. *Sf-9* cells were maintained in Graces medium supplemented with 10% FCS and 100 μg/ml antibiotic and antimycotic solution, and mouse lung endothelial cells were prepared and maintained as described previously (Boosani et al. 2007; Boosani and Sudhakar, 2006; Sudhakar et al. 2000). *Sf-9* insect cells were grown as monolayer cultures and maintained at 27 °C in complete medium. Only cells with greater than 95% viability were used for expression studies.

## Cloning, Expression and Purification of Mouse α3(IV)NC1 in *Sf-9* Cells

The sequence encoding mouse α3(IV)NC1 was PCR amplified using total RNA isolated from 129 Sv mouse kidney and SuperScript One-Step RT-PCR system supplemented with *Pfx* polymerase 5 units per reaction. The forward primer (5′-CGACATATGTCCTGGTGACAGGGGAACG-3′) and reverse primer (5′-TCTAGATCTCCAT-GTCTTTTCTTCATGCACACCT-3′) sequences were modified to incorporate *Nde* I and *Bgl* II restriction sites and were used to amplify a 720 bp encoding 240 amino acid sequence corresponding to α3(IV)NC1. PCR amplification was performed in PTC-100 Programmable Thermal Controller from MJ Research Inc, following the instructions in RT-PCR manual. The resulting amplicon was cloned into pBSIISKP vector at EcoR V site and the recombinant clones were identified by blue white selection. The clones were sequenced using T7 and T3 promoter primers (Table 1). The sequence confirmed clone was digested with *Nde* I and *Bgl* II to release the coding sequence corresponding to α3(IV)NC1. The released fragment was ligated into pAcHLT-A transfer vector (PharMingen) that was predigested with the same restriction enzymes, to generate recombinant viral transfer vector pAcHLT-A/α3(IV)NC1 that was used for co-transfection of *Sf-9* cells.

## Co-Transfection of *Sf-9* Cells

*Sf-9* cells were co-transfected with pAcHLT-A/α3(IV)NC1 viral transfer vector and linearized Baculogold^TM^ (Cat No: 21100D) viral DNA to obtain an infectious complete viral genome as reported earlier (Sudhakar et al. 2000; Boosani and Sudhakar, 2006).

## Plaque Assay and Amplification of Recombinant Virus

Plaque assay was carried out to identify recombinant virus in lysed co-transfected *Sf-9* cells. *Sf-9* cells were seeded at 1.8 × 10^6^ cells per 35 mm tissue culture dish. Several dilutions of recombinant virus ranging from 10^−1^ to 10^−7^ were made in 100 μl of complete medium and known viral dilution was added to each petridish and incubated at 27 °C for 1 hr with gentle rocking as reported previously by us (Sudhakar et al. 1999). Plaques were scored under a light microscope and the efficiency of the viral titer in terms of plaque forming units (pfu) was calculated: pfu/ml = Average No. of plaques X 1/ml of inoculums per plate X 1/dilution factor, as described previously (Boosani and Sudhakar, 2006).

## Dot-Blot Hybridization

Briefly, dot-blot hybridization was performed to identify the recombinant virus in which the 720 bp mouse α3(IV)NC1 cDNA has been incorporated into the viral genome. The radioactive probe corresponding to the α3(IV)NC1 was prepared and used to detect positive plaques with recombinant infectious virus. About 10^5^ *Sf-9* cells in 100 μl of medium were seeded into each well of a 96 well plate. One negative control with wild type virus (non-recombinant) and a positive control with the insert (template for the probe) were used as reported previously (Boosani and Sudhakar, 2006).

## *Sf-9* Cells Infection and Expression of Mouse α3(IV)NC1

Briefly, 4 × 10^6^ cells were seeded in T-25 tissue-culture flask and the cells were infected with about 1.6 × 10^8^ pfu/ml of α3(IV)NC1 viral titer for about 0–72 hrs. At each time point cells were washed with PBS and lysed in 200 μl of lysis buffer (20 mM Tris-HCl pH 7.8, 1 mM Mg^2+^, 1 mM DDT, pepstatin A (1 μg/ml), leupeptin (1 μg/ml), aprotinin (1 μg/ml) and PMSF 1 μM). After centrifugation at 13,000 rpm for 30 min, the clear cytosolic extracts were used for protein purification as described previously (Boosani and Sudhakar, 2006).

## Metal Affinity Purification of Mouse α3(IV)NC1

Recombinant mouse α3(IV)NC1 protein with 6xHis tag was expressed in *Sf-9* cells and cell lysate ~7 ml per batch (30 to 40 mg) was mixed with 1 ml of Ni-NTA Agarose affinity matrix. The extract and Ni-NTA Agarose was incubated for 1 hr at 4 °C on rocker and then centrifuged at 500 × g for 5 min at 4 °C. The supernatant was discarded and the 6xHis-tagged α3(IV)NC1 was eluted from the affinity matrix similar to our earlier reports (Boosani and Sudhakar, 2006). The fusion His tag was proteolytically cleaved from the recombinant protein at the thrombin cleavage site located upstream to the α3(IV)NC1 coding sequence. Recombinant mouse α3(IV)NC1 protein eluted from the affinity matrix was concentrated by 0%–80% ammonium sulphate fractionation, dialyzed against PBS and protein estimation was carried out using the Bio-Rad protein assay kit.

## Endothelial Cell Proliferation

A suspension of about 5.0 × 10^4^ HUVECs or MLEC cells/well, in a 24 well plate were used in proliferation assay. Cells were serum starved overnight and transferred to 24 well plates pre-coated with fibronectin and cultured for 24 hr in EGM-2 medium containing various concentrations of mouse α3(IV)NC1. After 24 hr, 1 μI of [^3^H]-thymidine was added into each well and further incubated at 37 °C for 24 hrs. Cell proliferation was assessed by studying the incorporation of [^3^H]-thymidine that was measured using scintillation counter as described previously (Sudhakar et al. 2005).

## Endothelial Cells Protein Synthesis

To study the effects on translation, protein synthesis in serum starved HUVECs or MLEC cells were studied. The cells were pre-incubated in methionine-free media for 1 hr and then labeled with radioactive methionine and further incubated for one more hour. The incorporation of radioactivity into trichloroacetic acid precipitates was analyzed after 48 hrs as described previously (Maeshima et al. 2002). ANOVA with a one tailed student’s *t* test was used to identify significant differences in multiple comparisons where p < 0.01 was considered statistically significant.

## 3-(4,5-Dimethylthiazol-2-yl)-2,5-Diphenyl Tetrazoliumbromide Assay (MTT) for Apoptosis

HUVECs or MLEC cells (8000 cells/well) were plated on a 96-well plate in EGM-2 medium. After 24 hrs, varying concentrations of α3(IV)NC1 was added and further incubated for 24 h. Cell viability was assessed by 3-(4,5-dimethylthiazol-2-yl)-2,5-diphenyl tetrazoliumbromide assay (MTT), as described previously (Boosani and Sudhakar, 2006).

## Migration Assay

About 10,000 HUVECs or MLEC cells in 30 μl of incomplete medium (ICM) with and without recombinant α3(IV)NC1 (1 μM) were seeded into each upper well of the Boyden chamber. In the lower wells of Boyden chamber, ICM containing 25 ng/ml bFGF was added and incubated for 24 hr at 37 ° C with 5% CO_2_. ICM alone was used as negative control (data not shown). The number of cells that migrated and attached to the bottom side of the membrane were counted as described previously (Boosani et al. 2007; Sudhakar et al. 2005).

## Tube Formation Assay

About 250 μl of matrigel-matrix was added to each well of a chilled 24 well plate and the matrix was allowed to polymerize for 30 min at 37 °C. A suspension of 5 × 10^4^ HUVECs or MLEC cells in EGM-2 without antibiotic were plated on top of the matrigel-matrix. Cells were treated with or without 1.0 μM α3(IV)NC1 and incubated for 48 hr at 37 °C, and the tube formation was observed using a CK2 Olympus microscope as reported earlier (Boosani et al. 2007; Sudhakar et al. 2005). All assays were performed in quadruplicates.

## Cell Signaling Experiments

About 10^6^ HUVECs or MLEC cells were seeded on a 10 cm^2^ petridish coated with fibronectin (10 μg/ml). The monolayer cell culture was pre-incubated with mouse α3(IV)NC1 or human end-ostatin and the cells were lysed with 200 μl of lysis buffer. The cell extracts were separated on a 10% SDS-PAGE and transferred onto a nitrocellulose membrane by western blotting. The signaling events were evaluated following immunoblotting using antibodies specific to phosphorylated and unphosphorylated proteins as described previously (Boosani et al. 2007; Sudhakar et al. 2005).

## *In vitro* Kinase Assay for mTOR Activity

Phosphorylation of mTOR and GST-4EBP-1 fusion protein (mTOR substrate) was evaluated in HUVECs or MLEC cells transfected with HA-mTOR/FRAP expression plasmid as described previously (Maeshima et al. 2002; Sudhakar et al. 2003). Briefly, cells were serum starved and transiently transfected with HA-mTOR/FRAP plasmid using Lipofectamine Plus reagent (Invitrogen, Carlsbad, CA). About 4 × 10^6^ transfected cells were treated with mouse α3(IV)NC1 and human end-ostatin (1 μM) for 24 hours according to the experimental protocol. The cells were lysed and 200 μg of extracts were subjected to immunoprecipitation with anti-HA antibody. HA-mTOR/Anti-HA complexes were incubated with recombinant GST-4E-BP1 fusion protein in presence of 10 μCi of [γ^32^P]-ATP in kinase buffer. The reactions were terminated by boiling, and the samples were subjected to SDS-PAGE. The phosphorylated proteins of mTOR-P and GST-4EBP1-P were detected by autoradiography.

## Results

### Expression and purification of mouse α3(IV)NC1 using baculovirus expression system

The sequence encoding mouse α3(IV)NC1 (720 bp) was amplified from 129 Sv mice kidney total RNA. The sequence was confirmed using high-throughput DNA sequencing facility at University of Nebraska Medical Centre, followed by a BLAST search that matched both the strands completely with published sequence of mouse collagen Type IV (Miner and Sanes, 1994). Comparing the mouse and human α3(IV)NC1 protein sequences showed 91% similarity at amino acid level, demonstrating a high degree of homology between them (mouse α3(V)NC1 sequence ACC number NM 007734 Vs human α3(V)NC1 sequence ACC number NM 000091).

**Figure 1:**
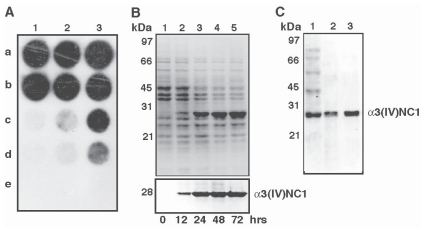
Cloning, expression and purification of mouse α3(IV)NC1. **Panel A.** Dot-blot analysis of the plaques containing recombinant virus expressing α3(IV)NC1: Plaques (a and b) lanes 1, 2 and 3 were found to be positive. The wells c and d (1 and 2) serve as negative controls containing non-recombinant AcNPV virus infected cell extracts, and well e (1, 2 and 3) corresponds to un-infected Sf9 cell extracts serves as negative control. α3(IV)NC1 cDNA was used as positive control in lane 3 (c and d). **Panel B.** Time course expression of α3(IV)NC1 in Sf9 insect cells at 12, 24, 48 and 72 hr of post infection: Lane 1 shows uninfected cell extracts at 0 hr (control). Each lane contains about 30 μg of crude cell extract and the figure is a coomassie-stained gel. Western immunoblot analysis of the time course expression of α3(IV)NC1 using α3(IV)NC1 antibody are shown in lower Panel. **Panel C.** Purification of α3(IV)NC1 using affinity matrix: Lane 1 shows 10 μg of flow through, lanes 2 and 3 shows 1 μg of affinity purified α3(IV)NC1 before and after dialysis.

The sequence encoding mouse α3(IV)NC1 was cloned into baculovirus transfer vector pAcHLT-A between Nde I and Bgl II restriction sites. The resulting recombinant baculovirus transfer vector pAcHLT-A/α3(IV)NC1 has the 6xHis tag located upstream to α3(IV)NC1 to enable one-step purification using affinity chromatography. The recombinant baculovirus viral transfer vector pAcHLT-A/α3(IV)NC1 was co-transfected with linearized BaculoGold^TM^ Baculovirus DNA to obtain an infectious virus as reported previously (Boosani and Sudhakar, 2006). To confirm the infectious complete viral genome harboring α3(IV)NC1, a radioactive probe corresponding to mouse α3(IV)NC1 cDNA was used to identify the recombinant plaques (Fig. 1A). Six out of 18 plaques used for α3(IV)NC1 were found positive (Fig. 1A lanes 1, 2 and 3 a, b). The supernatants of the positive plaques from the 96 wells were used for further amplification and expression as described previously (Boosani and Sudhakar, 2006; Sudhakar et al. 2000).

*Sf-9* cells were co-transfected with recombinant virus (MOI-10) harboring pAcHLT-A/α3(IV)NC1 and extracts were prepared at different time points of post-infection. Mouse α3(IV)NC1 was expressed as a 28 kDa soluble protein whose concentration increased with increase in post-infection time up to 72 hrs (Fig. 1B, lanes 2 to 5). Wild type AcNPV virus infected cells did not produce any protein of similar molecular mass (data not shown). The over expressed α3(IV)NC1 protein cross reacted with anti-α3(IV)NC1 antibody as observed through western immunoblotting (Fig. 1B lower panel). The purification profile of mouse α3(IV)NC1 using single step affinity matrix chromatography was carried out as described earlier (Boosani and Sudhakar, 2006) (Fig. 1C). After dialysis from the single step purification, the levels of expression was measured using BCA assay.

### Mouse α3(IV)NC1 has translation inhibition and anti-proliferative effects on endothelial cells

**Figure 2:**
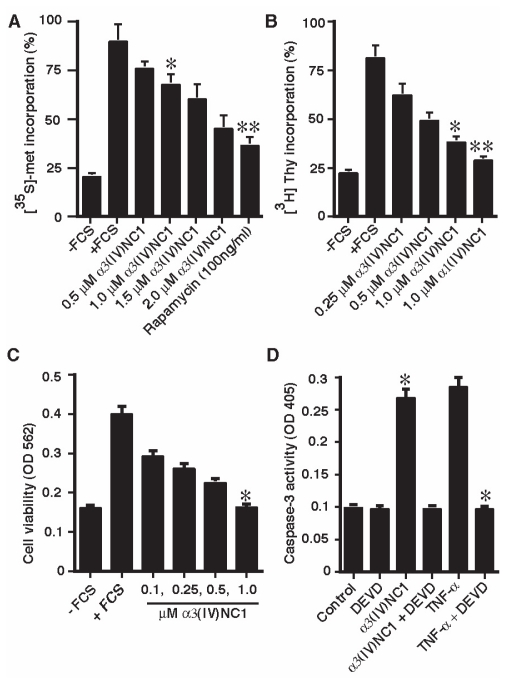
Functional characterization of mouse α3(IV)NC1. **Panel A.** The graph summarizes results from three independent protein synthesis experiments on the relative uptake of radioactive [^35^S] methionine at 48 hrs of α3(IV)NC1 treatment. *Indicates *P* < 0.03 (1 μM α3(IV)NC1 treatment compared to FCS. **Indicates *P* < 0.01 (rapamycin treatment compared to FCS). **Panel B.** The graph summarizes results from three independent proliferation experiments on the relative uptake of [^3^H]-thymidine incorporation. *Indicates *P* < 0.01 (1 μM α3(IV)NC1 treatment compared to FCS. **Indicates *P* < 0.005 (1 μM α1(IV)NC1 treatment compared to FCS). **Panel C.** Cell Viability assay: The 3-(4,5-dimethylthiazol-2-yl)-2,5-diphenyl tetrazoliumbromide assay (MTT) was used to evaluate HUVECs or MLEC cells viability after treatment with α3(IV)NC1. α3(IV)NC1 decreased the cell viability in a dose-dependent manner. *Indicates *P* < 0.005 (with and without 1 μM α3(IV)NC1 treatment. **Panel D.** Control and α3(IV)NC1 treated cells were lysed, and caspase-3 activity was detected. DEVD and TNF-α were used as positive controls. *Indicates *P* < 0.005 (TNF-α alone or with TNF-α with DEVD treatment. In panel A-C −FCS and +FCS represents cells grown in 0.1% and 10% FCS medium.

HUVECs or MLEC cells showed reproducible translation and proliferation inhibition response for human α3(IV)NC1 (Maeshima et al. 2002). We initially used these cells to examine the translation inhibitory action and anti-proliferative effects of mouse α3(IV)NC1. As expected, we observed dose dependent inhibitory effect on protein synthesis in endothelial cells upon mouse α3(IV)NC1 treatment. About 2 μM concentration of mouse α3(IV)NC1 showed 50% inhibition of protein synthesis (Fig. 2A). Similar inhibition of protein synthesis was also reported previously using human α3(IV)NC1 (Maeshima et al. 2002). These results conform that mouse α3(IV)NC1 shows protein synthesis inhibition similar to human α3(IV)NC1. Further we also observed dose dependent inhibition of proliferation upon mouse α3(IV)NC1 treatment to endothelial cells (Fig. 2B). Interestingly, mouse α3(IV)NC1 did not show any effect on translation or proliferation of human 789-0 renal cell carcinoma and LLC cells (data not shown).

### Induction of apoptosis in endothelial cells by mouse α3(IV)NC1

In order to understand the mechanism of action of mouse α3(IV)NC1 on endothelial cells, first we studied whether the inhibitory action in endothelial cells was due to its effect on cell viability, and thus treated HUVEC or MLEC cells with different concentrations of mouse α3(IV)NC1. A dose dependent cell death was observed with increasing concentrations of mouse α3(IV)NC1 treatment (Fig. 2C). To further understand the endothelial cell death mediated signaling by α3(IV)NC1, we carried out caspase-3 activation assays. Caspase-3 is an intracellular protease activated at the early stages of apoptosis and initiates cellular breakdown by degrading structural and DNA repair proteins (Magnon et al. 2005). The caspase-3 activity was measured spectrophotometrically through the detection of chromophore (p-nitroanilide) cleaved from the labeled substrate (DEVD-p-nitroanilide) upon α3(IV)NC1 treatment in endothelial cells. α3(IV)NC1 treated endothelial cells exhibited a 4 fold increase in caspase-3 activity, whereas TNF-α a known caspase-3 activator treatment gave 4.6 fold increase of caspase-3 activity compared to control (Fig. 2D). A specific inhibitor of caspase-3, DEVD, decreased caspase-3 activity to baseline indicating that the increase in the measured activity was specific for caspase-3. These results suggests that one of the mechanisms by which mouse α3(IV)NC1 affects cell proliferation is through caspase-3 activation.

### Antiangiogenic effects of mouse α3(IV)NC1

**Figure 3:**
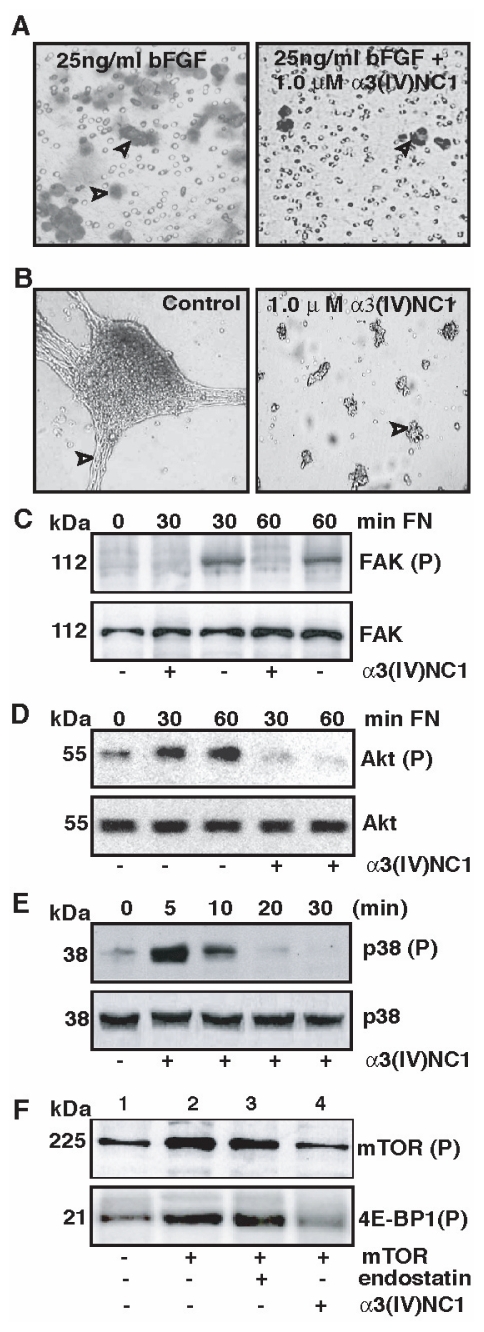
Cell signaling experiments with mouse α3(IV)NC1. **Panel A.** Migration of HUVECs or MLEC cells with and without mouse α3(IV)NC1. **Panel B.** Tube formation of endothelial cells on Matrigel matrix with and without α3(IV)NC1. Migration and tube formation of endothelial were viewed using a light microscope at 100x magnification. **Panel C-E.** Serum-starved HUVEC or MLEC cells were plated on fibronectin coated culture plate supplemented with 1 μ α3(IV)NC1 for the indicated times and cytosolic extracts were analyzed by western blotting. Immunoblots of phosphorylated FAK or Akt or p38 (top blot) and total FAK or Akt of p38 (lower blot) were shown. **Panel F.** mTOR kinase Assay: Autoradiograph of the autophosphorylated mTOR (top blot) and phosphorylated 4E-BP1 (lower) isolated from (HA)-mTOR transfected HUVECs shown. (P) and FN represents phosphorylated protein and fibronectin.

Next we utilized mouse α3(IV)NC1 protein to test the migratory effect on HUVECs or MELC cells across fibronectin coated membrane towards bFGF in a Boyden chamber. Surprisingly, we identified the anti-migratory effect of mouse α3(IV)NC1 similar to other collagen NC1 domain which we have previously reported (Sudhakar et al. 2005) (Fig. 3A). These results supports that mouse α3(IV)NC1 is capable of inhibiting endothelial cell migration, where as human molecule is not (Boosani et al. 2003). Our results, suggests that mouse α3(IV)NC1 is a more potent anti-angiogenic molecule compared to human α3(IV)NC1. In addition antiangiogenic activity of mouse α3(IV)NC1 was confirmed by tube formation assay. Addition of mouse α3(IV)NC1 protein to the endothelial cell culture media significantly inhibited tube formation on matrigel matrix (Fig. 3B). Our earlier reports also indicate that human α3(IV)NC1 inhibits tube formation in endothelial cells (Boosani et al. 2007; Sudhakar et al. 2003).

### Signal transduction cascades induced by recombinant mouse α3(IV)NC1

Finally, the characteristic antiangiogenic activity of mouse α3(IV)NC1 was confirmed by integrin mediated cell signaling experiments. In endothelial cells, ligand upon binding to integrins induces phosphorylation of focal adhesion kinase (FAK), which serves as a platform for different downstream signals (Zachary and Rozengurt, 1992). Recombinant mouse α3(IV)NC1 inhibited phosphorylation of FAK when endothelial cells were cultured on fibronectin (Fig. 3C). Downstream to FAK, protein kinase B (PKB/Akt), phosphatidyl-3-kinase (PI3 kinase) plays an important role in mediating pathways that are involved in the regulation of endothelial cell survival (Maeshima et al. 2002; Shiojima and Walsh, 2002; Sudhakar et al. 2003). Akt was also known to regulate protein synthesis mediated by phosphorylation of eukaryotic initiation factor 4E-binding protein (4E-BP1) via mTOR kinase (Miron et al. 2001).

Our studies show that mouse α3(IV)NC1 inhibited sustained phosphorylation of Akt activation and p38 MAP kinase in HUVECs or MLEC cells that are plated on fibronectin (Fig. 3, D and E). Downstream to Akt, mTOR directly phosphorylates eIF4E-binding protein (4E-BP1) and unphosphorylated 4E-BP1 interacts with eIF4E and inhibits cap-dependent protein synthesis (Maeshima et al. 2002; Pause et al. 1994; Sudhakar et al. 2003). Recombinant mouse α3(IV)NC1 suppressed mTOR activity and inhibited phosphorylation of 4E-BP1 in endothelial cells similar to human α3(IV)NC1 (Fig. 3F). Inhibition of 4E-BP-1 phosphorylation enhanced binding to eIF-4E, which in turn becomes unavailable to initiate cap-dependent translation (Fig. 3F, lower blot).

### Effect of α3(IV)NC1 on SCC-PSA1 tumor growth and endothelial cell apoptosis

**Figure 4:**
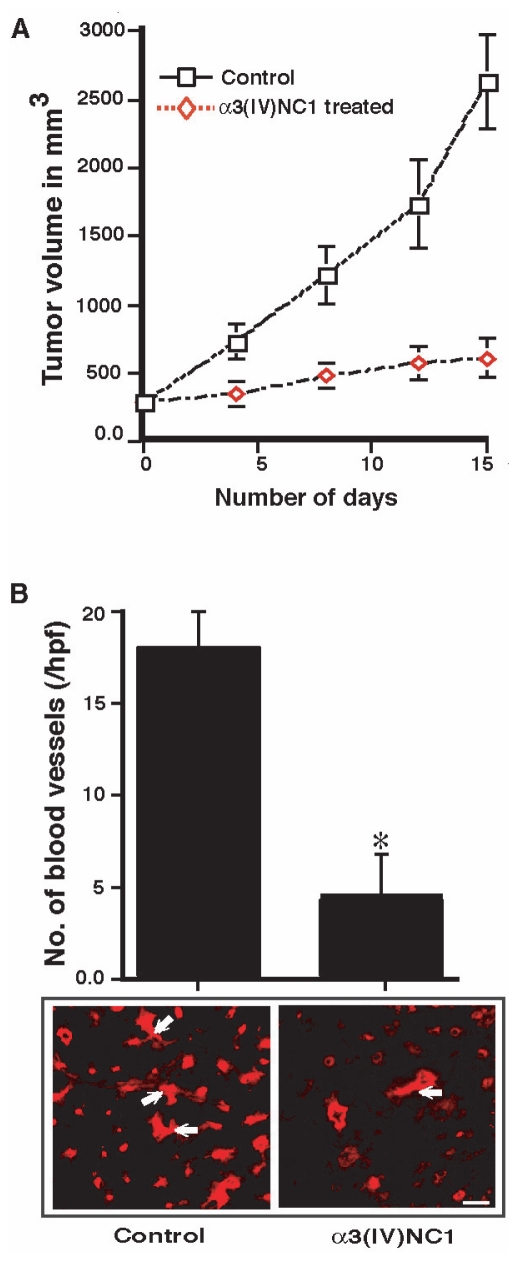
Inhibition of tumor growth and tumor angiogenesis in 129/Sv mice. **Panel A.** We injected mouse α3(IV)NC1 protein to SCC-PSA1 tumor bearing mice daily for 15 days. Data are representative of three such independent experiments. The results are shown as the mean ± SEM and p < 0.001 compared to mice with and without α3(IV)NC1 injection. **Panel B.** Frozen sections (4-μm) from tumor tissue were stained with anti-CD31 antibody and the number of CD31 positive blood vessels were counted in 6 fields. The blood vessel quantification results were shown as the mean ± SEM. *Indicates p < 0.001; compared to mice with and without mouse α3(IV)NC1 treatment. Scale bar corresponds to 50 μM. Arrows indicated CD31 positive endothelial vasculature.

We examined the effect of mouse α3(IV)NC1 on SCC-PSA1 (teratocarcinoma) tumor models in 129Sv mice. Treatment of mouse α3(IV)NC1 at 30 μg concentration showed significant inhibitory effect on SCC-PSA1 tumor growth similar to human α3(IV)NC1 recently reported by us (Fig. 4A) (Boosani et al. 2007). The number of CD-31 positive blood vessels in α3(IV)NC1 treated tumors were significantly inhibited compared to control tumors (Fig. 4B).

Collectively, our results suggested that baculovirus expressed mouse α3(IV)NC1 inhibits FAK/Akt activation and leads to inhibition of p38/mTOR/4E-BP1 and cap-dependent translation with significant effect on migration, whereas human α3(IV)NC1 did not show any effect on endothelial cell migration and p38 MAPK phosphorylation. Further analysis of this molecule and its role in antiangiogenesis and cancer needs extensive evaluation.

## Discussion

A number of endogenous, endothelial cell-specific angiogenesis inhibitors have been identified and were functionally characterized both *in vitro* and *in vivo*. Many of these molecules were found to be shorter fragments of large parent molecules generated by proteases and gaining new properties. For example, endostatin and several type IV collagen non-collagenous (NC1) domains show antiangiogenic activities (O’Reilly et al. 1997; Petitclerc et al. 2000). Among type IV collagen NC1 domains, α3(IV)NC1 is extensively studied for its anti-angiogenic activity (Boosani et al. 2007; Borza et al. 2006; Maeshima et al. 2002; Petitclerc et al. 2000). Mouse α3(IV)NC1 was found inhibiting endothelial cell migration and proliferation, where as human α3(IV)NC1 is inhibiting endothelial proliferation but not migration (Sudhakar et al. 2003). Therefore large scale production of soluble mouse α3(IV)NC1 is needed to better understand its *in vivo* mechanism (s) of action and for its potential therapeutic use.

In this study, we demonstrate for the first time that biologically active mouse α3(IV)NC1, can be expressed *in Sf-9 cells* using baculovirus expression system. Mouse α3(IV)NC1 was expressed as a soluble form (28 kDa) and purified using single step protocol (Boosani and Sudhakar, 2006). The yield was significantly improved compared to human α3(IV)NC1 in 293 human kidney cells and the purification process was very economical. However, in the baculovirus system several factors significantly improve the yield (>2 to 3 fold) of expressed proteins such as viral titer, cell viability, temperature etc. Using a similar baculovirus expression system we recently demonstrated efficient production of human α1(IV)NC1 (Boosani and Sudhakar, 2006). Baculovirus expressed recombinant α3(IV)NC1 protein was soluble and was biologically active in a variety of *in vitro* and *in vivo* experiments. Recombinant mouse α3(IV)NC1 inhibited proliferation and translation of HUVECs or MLEC cells similar to human α3(IV)NC1. Both mouse and human α3(IV)NC1 showed similar effects on endothelial cell viability and apoptosis in a dose dependent manner.

Recently we reported that human α3(IV)NC1 regulates cap dependent translation and tumor angiogenesis in a COX-2 dependent manner (Boosani et al. 2007; Maeshima et al. 2002; Sudhakar et al. 2003). Our data supports that mouse α3(IV)NC1 effects FAK/Akt/mTOR/4E-BP1 signaling similar to human NC1. Whereas mouse α3(IV)NC1 shows specific differences in functional activities compared to human α3(IV)NC1 such as inhibition of endothelial cell migration and p38 MAPK phosphorylation (Table 2). Human and mouse α3(IV)NC1 share 91% sequence homology, whereas mouse α3(IV)NC1 lacks RGD sequence in the N terminal end. Whether apoptosis and proliferation is a prerequisite for the cap-dependent translation inhibition by human α3(IV)NC1 remains to be explored. Thus the availability of this protein in its biologically active form in large scale (quantities) will helps further functional analysis of this protein.

## Figures and Tables

**Table 1:**
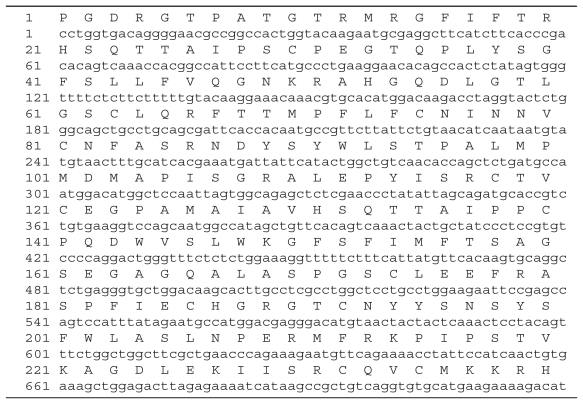
Mouse a3(IV)NC1 sequence (720 bp)

**Table 2:** Distinct signaling mechanisms of mouse and human α3(IV)NC1.

	Mouse α3(IV)NC1	Human α3(IV)NC1
Origin	α3Type IV collagen	α3 Type IV collagen
Endothelial proliferation	Inhibition	Inhibition
Endothelial migration	Inhibition	No effect
Endothelial tube formation	Inhibition	Inhibition
Endothelial specific mechanism of action	FAK, Akt, p38, PI3 kinase, mTOR, eIF-4E/4E-BP1 mediated signaling and apoptosis	FAK, Akt, PI3 Kinase, mTOR, eIF-4E/4E-BP1 and COX-2 mediated signaling

## Keywords

α3(IV)NC1: non-collagenous α3 chain of type IV collagen
AcNPV: Autographa californica nuclear polyhedrosis virus
Sf-9: Spodoptera frugiperda
MLEC: mouse lung endothelial cells
HUVEC: human umbilical vein endothelial cells
FCS: fetal calf serum
PMSF: Phenylmethylsulfonyl Fluoride
TNF-α: tumor necrosis factor alpha
DEVD: cell membrane permeable caspase inhibitor
NC: negative control
PC: positive control
FN: fibronectin
MAPK: mitogen activated protein kinase
FAK: focal adhesion kinase
PKB/Akt: protein kinase B
mTOR: mammalian target of rapamycin
4E-BP1: eukaryotic initiation factor 4E-binding protein
